# Cerebral Blood and Cerebrospinal Fluid Flow Dynamics in Endurance Athletes: Associations With Aortic Recoil and Heart Rate

**DOI:** 10.1111/sms.70261

**Published:** 2026-03-12

**Authors:** Daisuke Hoshi, Marina Fukuie, Tsubasa Tomoto, David C. Zhu, Rong Zhang, Keigo Ohyama‐Byun, Seiji Maeda, Jun Sugawara, Takashi Tarumi

**Affiliations:** ^1^ Human Informatics and Interaction Research Institute National Institute of Advanced Industrial Science and Technology Tsukuba Japan; ^2^ Japan Society for the Promotion of Science Tokyo Japan; ^3^ Department of Sports Sciences Japan Institute of Sports Sciences Tokyo Japan; ^4^ Integrated Research Center for Self‐Care Technology National Institute of Advanced Industrial Science and Technology Tsukuba Japan; ^5^ Department of Radiology and Gruss Magnetic Resonance Research Center Albert Einstein College of Medicine Bronx New York USA; ^6^ Institute for Exercise and Environmental Medicine Texas Health Presbyterian Hospital Dallas Dallas Texas USA; ^7^ Department of Neurology University of Texas Southwestern Medical Center Dallas Texas USA; ^8^ Department of Internal Medicine University of Texas Southwestern Medical Center Dallas Texas USA; ^9^ Institute of Health and Sport Sciences University of Tsukuba Ibaraki Japan; ^10^ Sports Physiology, School of Sport Sciences Waseda University Saitama Japan

**Keywords:** aortic recoil, cerebral blood flow, cerebrospinal fluid, endurance athlete, Monro‐Kellie doctrine, Windkessel function

## Abstract

Endurance training elicits profound cardiovascular adaptations, including lower heart rate (HR), greater stroke volume (SV), and enhanced aortic Windkessel function. This study aimed to investigate cerebral blood and cerebrospinal fluid (CSF) flow dynamics in endurance athletes and their relations with cardiovascular parameters. Fifteen young male endurance athletes were compared with 19 age‐matched sedentary male controls. In the resting supine condition, CINE phase‐contrast MRI acquired the extra‐ and intracranial cerebral blood and CSF flow waveforms, as well as HR, SV, and ascending aortic cross‐sectional area waveform. Cerebral blood and CSF flow volume (mL/beat) and rate (mL/min) were quantified for the full cardiac cycle and separately for systolic and diastolic phases. Aortic cross‐sectional area change was temporally integrated to quantify aortic expansion and recoil. Athletes exhibited higher aortic recoil (5.7 cm^2^·s [4.1, 7.3] vs. 4.1 cm^2^·s [2.6, 5.6]) and SV (97 ± 18 mL vs. 82 ± 11 mL) and lower HR (53 ± 6 bpm vs. 67 ± 12 bpm) than controls. Total cerebral arterial blood and CSF flow volumes and rates across the full cardiac cycle were similar between groups; however, athletes showed significantly greater diastolic arterial flow volume and lower systolic arterial flow rate. The association between lower HR and greater diastolic arterial flow volume was mediated by higher aortic recoil across all participants. These results suggest that enhanced aortic recoil in endurance athletes sustains diastolic cerebral arterial flow volume across a prolonged cardiac cycle, enabling more efficient brain perfusion with fewer cardiac cycles than sedentary controls.

## 1 Introduction

The brain has high metabolic demands but limited intrinsic energy reserves. Thus, a continuous supply of oxygen and nutrients via the cardiovascular system is essential for maintaining normal neuronal function [1, 2]. This high metabolic activity also produces substantial metabolic waste that must be efficiently cleared to preserve microenvironmental homeostasis [3, 4]. Cerebrospinal fluid (CSF) plays a critical role in this process by facilitating fluid exchange between CSF and interstitial fluid and promoting waste clearance through venous and lymphatic pathways [5, 6]. One of the primary driving forces for cerebral blood and CSF flow is arterial pulsatility generated by the left ventricular ejection of stroke volume (SV) [5]. The arterial pressure and flow pulsatility are dampened by the elastic central arteries such as the aorta and carotid arteries, and transmitted into the intracranial space that is enclosed by a rigid skull [7].

Endurance training induces profound cardiovascular adaptations, including lower resting heart rate (HR) and higher SV at rest and during submaximal exercise [8]. While these adaptations can enhance the capacity to augment the cardiac output during high‐intensity endurance exercise, increased SV could theoretically amplify arterial pulsatility transmitted to the brain, potentially imposing mechanical stress on the cerebral microvasculature. In addition, prolonged cardiac cycles associated with bradycardia may increase the risk of cerebral hypoperfusion during diastole. On the other hand, our recent work has shown that endurance athletes exhibit marked structural and functional adaptations of the proximal aorta, including larger cross‐sectional area, enhanced compliance, and reduced input impedance compared with sedentary controls [9, 10]. These changes indicate enhanced aortic Windkessel function in endurance athletes, which may buffer the excessive pulsatility from greater SV while augmenting diastolic cerebral perfusion through elastic recoil [11]. Thus, the aortic adaptations to endurance training may play pivotal roles in efficient cerebral perfusion in athletes through increasing the relative contribution of diastole to cerebral blood flow. However, the implications of enhanced aortic Windkessel function for cerebral blood and CSF flow dynamics in endurance athletes remain unclear.

According to the Monro‐Kellie doctrine, the total volume of brain tissue, blood, and CSF within the rigid cranial cavity remains constant, thereby stabilizing intracranial pressure [12]. Across the cardiac cycle, systolic arterial inflow is balanced by venous and caudal CSF outflow, whereas diastolic phase is characterized by reduced arterial inflow accompanied by diminished venous outflow and increased cranial CSF inflow [13, 14]. These dynamic interactions between cerebral blood and CSF flow within each cardiac cycle can be quantified by time‐resolved (CINE) phase‐contrast magnetic resonance imaging (PC‐MRI) [15]. Using this imaging technique, we previously reported acute exercise effects on cerebral blood‐CSF flow interactions [16]; however, the impact of chronic cardiovascular adaptations to endurance training (i.e., lower HR, greater SV, and enhanced aortic recoil) on cerebral flow dynamics has not been investigated.

The present study aimed to compare cerebral blood and CSF flow dynamics between endurance‐trained athletes and age‐ and sex‐matched sedentary individuals and to further explore the role of aortic recoil in cerebral flow dynamics. We hypothesized that (1) endurance athletes characterized by slower HR, greater SV, and enhanced aortic recoil would exhibit a higher proportion of diastolic to systolic cerebral arterial flow volume across the cardiac cycle compared with sedentary individuals, and (2) higher aortic recoil would mediate the associations of greater diastolic cerebral arterial flow volume with slower HR and greater SV.

## 2 Materials and Methods

### 2.1 Participants

Fifteen young male endurance athletes and 19 age‐matched male sedentary adults were enrolled. Athletes were recruited from the University of Tsukuba track‐and‐field team and were actively training for middle‐ and long‐distance events. Their regular training included 12 sessions (~60 min each) per week: four high‐intensity interval runs, four moderate‐intensity continuous runs, and four low‐intensity jogging sessions. They also performed strength training (e.g., bench press, squat, deadlift, snatch) twice per week for ~30 min per session. Sedentary participants were recruited through community‐based advertisements on a local website, with the inclusion criterion being no participation in structured exercise or physical activity programs for at least the past 3 years. Exclusion criteria for both groups included history of cardiovascular, cerebrovascular, renal, or neurological disorders; cigarette smoking; use of cardiovascular‐acting medications (e.g., antihypertensives, antilipidemics, antidiabetics); claustrophobia; or the presence of metal implants incompatible with MRI. The study protocol was approved by the Institutional Review Board of the National Institute of Advanced Industrial Science and Technology (Hito 2018–873) and conducted in accordance with the Declaration of Helsinki and the Belmont Report. All participants provided written informed consent.

Some data from this cohort were reported in our previous studies examining the proximal aortic compliance [9], cerebral white matter microstructure [17], and aortic input impedance [10]. However, the current study has a different aim from the previous studies, which focus specifically on cerebral blood and CSF flow dynamics in endurance athletes versus sedentary controls.

### 2.2 Study Protocol

Participants were instructed to fast for at least 2 h and abstain from caffeine, alcohol, and strenuous exercise for over 24 h before the visit. Upon arrival, they completed medical history, physical activity, and MRI safety questionnaires. Height and body mass were measured to calculate body mass index (BMI). After 10 min of seated rest, brachial systolic blood pressure (SBP), diastolic blood pressure (DBP), and HR were measured at least twice using an automated monitor (HEM‐7130; Omron Corporation, Kyoto, Japan).

Subsequently, brain and cardiac MRI scans were performed in the supine position using a 3‐Tesla scanner (Ingenia, Philips Medical Systems, Netherlands) equipped with 32‐channel head and body coils (dStream). HR and respiration were continuously monitored via wireless electrocardiogram and a peripheral pulse unit (Invivo Corporation, USA), which provided cardiac gating for CINE PC‐MRI. Aortic and brachial BPs were recorded during MRI using the SphygmoCor XCEL system, following the previously established protocols [9, 10, 16]. Maximal oxygen uptake (VO_2_max) was measured in athletes on a separate day. Left ventricular morphometry (*n* = 16; 6 athletes and 10 sedentary individuals) and brain volumetrics (*n* = 32; 15 athletes, 17 sedentary individuals) were measured in subgroups, as reported in our previous studies [9, 10, 17].

### 2.3 Data Collection and Analysis

#### 2.3.1 Cerebral Blood and CSF Flow Measurements

**FIGURE 1:**
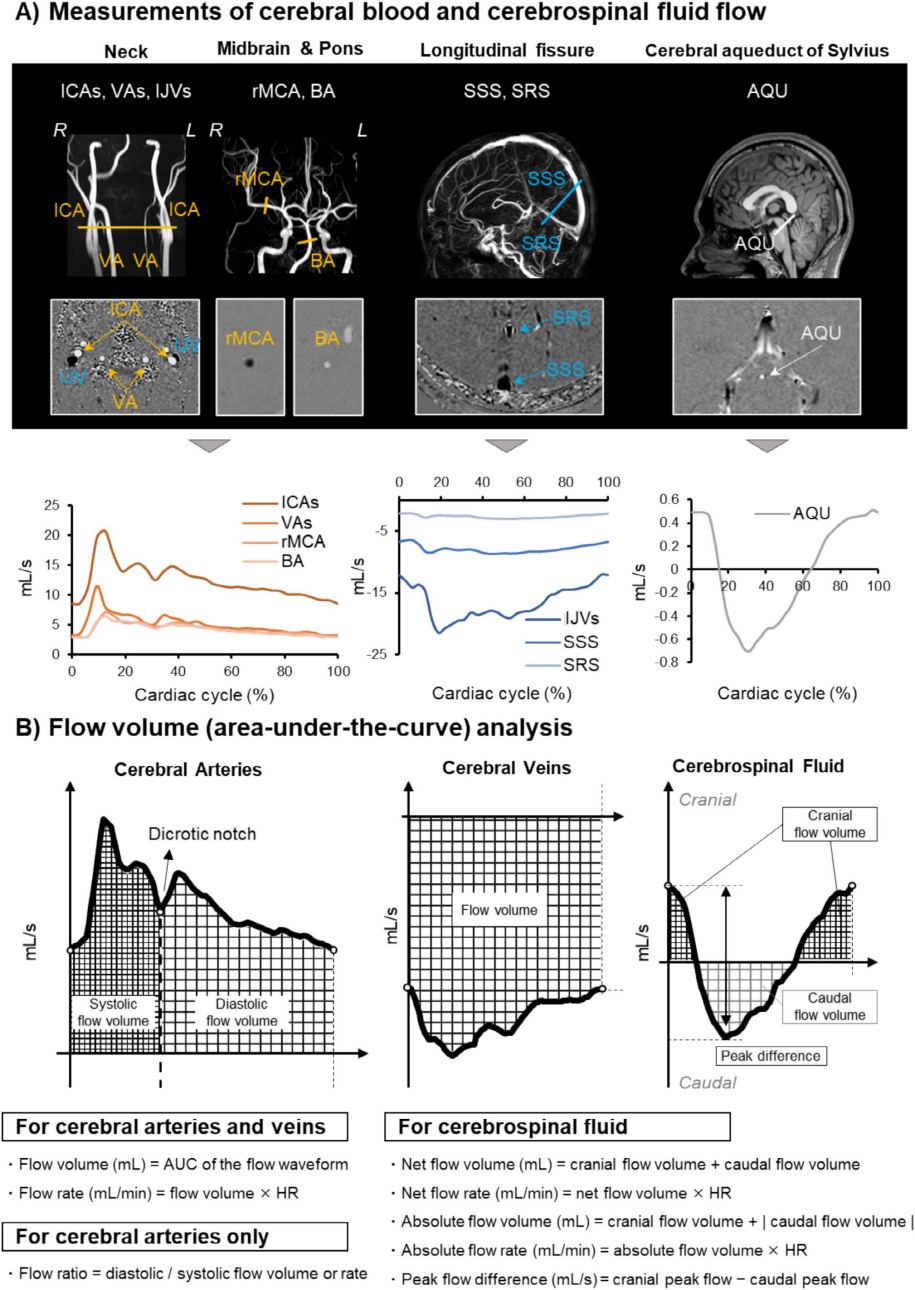
(A) Slice locations for time‐resolved (CINE) phase‐contrast magnetic resonance imaging (PC‐MRI). Cerebral arterial, venous, and cerebrospinal fluid (CSF) flow waveforms were acquired separately at the orange, blue, and white lines, respectively. Blood flow was measured bilaterally in the internal carotid (ICAs) and vertebral (VAs) arteries and internal jugular veins (IJVs), as well as in single unilateral vessels including the basilar artery (BA), right middle cerebral artery (rMCA), superior sagittal sinus (SSS), and straight sinus (SRS). CSF flow was measured in the cerebral aqueduct of Sylvius (AQU). Phase images and original flow waveforms of the corresponding vessels and aqueduct are shown for one representative athlete. Cranially and caudally directed flows are represented by bright‐white and dark‐gray pixels and plotted as positive and negative flow values, respectively. (B) Area‐under‐the‐curve (AUC) analysis of arterial, venous, and CSF flow waveforms. Flow volume was quantified across the full cardiac cycle and separately for systolic and diastolic phases, divided by the dicrotic notch on the arterial waveform.

As shown in Figure 1A, slice locations for CINE PC‐MRI measurements were determined using time‐of‐flight neck and midbrain angiograms, a sagittal venogram along the longitudinal fissure, and sagittal T1‐ and T2‐weighted images. A total of five separate CINE PC‐MRI scans were acquired at the following locations. (1) At the neck, extracranial arterial and venous flows were measured bilaterally from the internal carotid arteries (ICAs) and vertebral arteries (VAs) and the internal jugular veins (IJVs). (2) Right middle cerebral arterial (rMCA) flow was measured at the proximal M1 segment. (3) Basilar arterial (BA) flow was measured at the straight, mid‐pons midline level angulated orthogonal to basilar flow. (4) Intracranial sinus flow was measured from the superior sagittal sinus (SSS) and straight sinus (SRS) in the occipital area. (5) CSF flow was measured at the cerebral aqueduct of Sylvius, which is a major CSF pathway located close to the ventricles and choroid plexus, with its narrow, well‐defined structure reflecting intracranial compliance and pressure transmission [18]. Based on previous studies in young adults reporting comparable blood flow between the right and left MCAs as well as between the extracranial and intracranial ICAs [19], the left MCA and intracranial ICA were excluded from the imaging vessels. Phase images of the corresponding vessels and aqueduct are presented in Figure 1A. Detailed region‐of‐interest (ROI) selection procedures are provided in the Supporting Information.

All CINE PC‐MRI scans were performed using a 2D fast field‐echo phase‐contrast sequence with retrospective cardiac gating via a peripheral pulse unit to reconstruct 32 cardiac phases across the cardiac cycle. The acquisition parameters for CINE PC‐MRI were as follows:

- ICAs, VAs, and IJVs: field‐of‐view (FOV) = 150 × 150 mm^2^, acquisition matrix = 132 × 130, in‐plane resolution = 1.15 × 1.15 mm^2^ (reconstructed voxel size = 0.59 × 0.59 mm^2^), slice thickness = 5 mm, repetition time (TR) = 9 ms, echo time (TE) = 5 ms, flip angle = 10°, velocity encoding (VENC) = 100 cm/s, and scan duration ≈4 min.
- BA and rMCA*: FOV = 150 × 101 mm^2^, acquisition matrix = 132 × 88, in‐plane resolution = 1.15 × 1.15 mm^2^ (reconstructed voxel size = 0.59 × 0.59 mm^2^), slice thickness = 5 mm, TR = 9 ms, TE = 5 ms, flip angle = 10°, VENC = 120 cm/s, and scan duration ≈3 min. *The same parameters were used for both vessels.
- SSS and SRS: FOV = 150 × 150 mm^2^, acquisition matrix = 132 × 130, in‐plane resolution = 1.15 × 1.15 mm^2^ (reconstructed voxel size = 0.59 × 0.59 mm2), slice thickness = 5 mm, TR = 9 ms, TE = 5 ms, flip angle = 10°, VENC = 60 cm/s, and scan duration ≈4 min.
- Cerebral aqueduct of Sylvinus: FOV = 150 × 150 mm^2^, acquisition matrix = 256 × 179, in‐plane resolution = 0.59 × 0.84 mm^2^ (reconstructed voxel size = 0.59 × 0.59 mm^2^), slice thickness = 5 mm, TR = 12 ms, TE = 8 ms, flip angle = 10°, VENC = 15 cm/s, and scan duration ≈7 min.

Scan duration varied depending on participants' HR. Through‐plane velocities were recorded with flow compensation to reduce image flow artifacts. All scans were visually inspected, and no velocity aliasing was observed.

CINE PC‐MRI data were analyzed using Q‐flow software (Philips Medical Systems, Netherlands) as described in previous studies [9, 10, 16]. ROIs were manually defined at peak systole on magnitude images to encompass the full vessel or aqueduct cross‐section and then automatically propagated through all cardiac phases. Cerebral blood and CSF flow waveforms of a single cardiac cycle were calculated as the product of velocity and cross‐sectional area (CSA). The cranial (arterial and CSF inflow) and caudal (venous and CSF outflow) flows were expressed as positive and negative values, respectively.

#### 2.3.2 Flow Waveform Analysis

To preserve cine‐defined cardiac phase timing and allow phase‐specific comparisons without waveform stretching or truncation, the original 32‐point waveform was resampled at 100 Hz using the AcqKnowledge software (version 5.0; BIOPAC Systems, Santa Barbara, CA, USA). Flow parameters were subsequently computed for each ROI (Figure 1B). In this study, cerebral blood and CSF flow measured by CINE PC‐MRI are expressed as volumetric flow per beat (flow volume, mL) and flow per minute (flow rate, mL/min) as follows:

- Flow volume (mL) = area‐under‐the‐curve (AUC) of the flow waveform.
- Flow rate (mL/min) = flow volume × HR.
- Flow ratio = diastolic flow volume or rate/systolic flow volume or rate.

Arterial and venous flow volume and rate were calculated for the full cardiac cycle. Additionally, arterial flow measures were calculated during systolic and diastolic phases that were separated by the dicrotic notch [20], identified via first derivative and visual inspection of flow waveforms. To assess relative contributions, arterial flow ratio was calculated as diastolic flow divided by systolic flow. Bilateral flows were summed to reduce the number of comparisons, and total cerebral arterial flow was defined as ICA + VA flow.

In contrast to blood flow, CSF flow is bidirectional consisting of caudal flow during systole (driven by arterial inflow) and cranial flow during diastole. Thus, CSF flow metrics were calculated by the following equations based on the previous study [16]:

- Net flow volume (mL) = cranial flow volume + caudal flow volume.
- Net flow rate (mL/min) = net flow volume × HR.
- Absolute flow volume (mL) = cranial flow volume + | caudal flow volume |.
- Absolute flow rate (mL/min) = absolute flow volume × HR.
- Peak flow difference (mL/s) = cranial peak flow − caudal peak flow.

where absolute flow values reflect total CSF pulsatility, while net values describe overall unidirectional CSF movement.

Additionally, linear slope analysis quantified the pulsatile waveform morphology by connecting predefined waveform landmarks within one cardiac cycle (Figure S1). Pulsatility index (PI) was also calculated for the arterial and venous blood flow using Gosling's formula [21]. For CSF, peak flow difference was used as a surrogate of arterial and venous PIs as net flow volume is close to zero (Figure 1B).

### 2.4 Aortic Recoil

**FIGURE 2:**
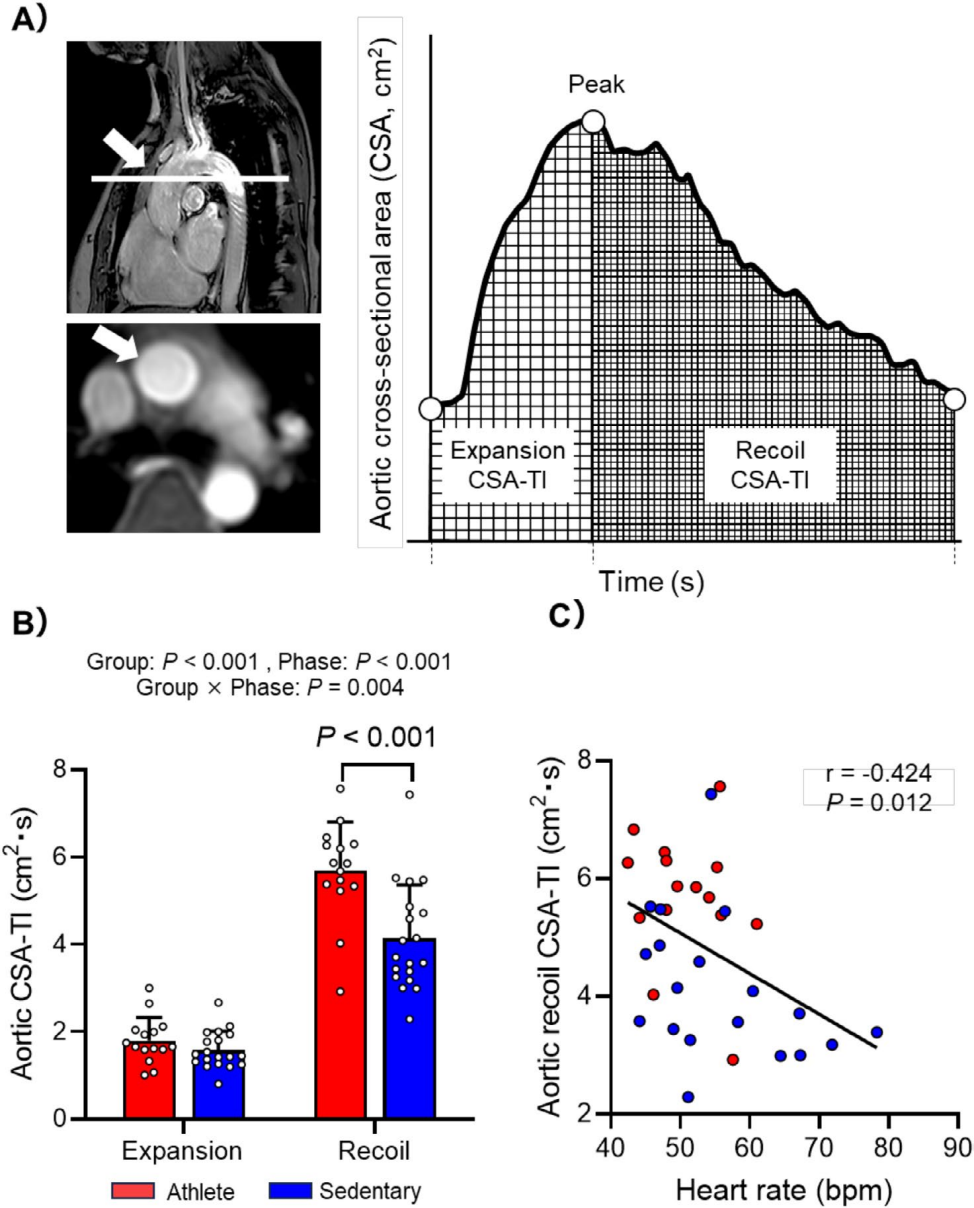
(A) Cross‐sectional area (CSA) waveform of the ascending aorta (arrow) across the cardiac cycle measured by time‐resolved (CINE) phase‐contrast magnetic resonance imaging. The aortic CSA‐time integral (CSA‐TI) was calculated for the expansion and recoil phases. (B) Comparison of aortic CSA‐TI during expansion and recoil phases between athlete and sedentary groups. *p*‐values were derived from linear mixed model analysis. (C) Simple correlation between heart rate and aortic recoil CSA‐TI in all participants.

To assess the influence of aortic wall recoil on cerebral hemodynamics, the CSA‐time integral (CSA‐TI) of the ascending aorta was calculated during expansion and recoil phases (Figure 2A left). As described previously [9, 10], CINE PC‐MRI scans of the ascending aorta were acquired above the pulmonary artery bifurcation with the following parameters: FOV = 350 × 300 mm^2^, acquired in‐plane resolution = 2.5 × 2.5 mm^2^ (reconstructed voxel size = 1.21 × 1.21 mm^2^), slice thickness = 8 mm, TR = 4 ms, TE = 2.4 ms, flip angle = 10°, VENC = 180 cm/s, number of signal acquisitions = 2, and total scan duration = ∼5 min depending on HR. Using the Q‐flow software, CSA waveforms of the ascending aorta were generated, from which CSA‐TI during expansion and recoil was derived using peak CSA as a boundary (Figure 2A right). SV and cardiac output were also measured from the aortic CINE PC‐MRI data.

### 2.5 Brain Tissue, CSF, and Intracranial Volumes

Volumetric brain measures were calculated from T1‐weighted 3D MPRAGE images acquired with the following parameters [17]: FOV = 256 × 256 mm^2^, 176 sagittal slices (no gap), acquired and reconstructed voxel size = 1 mm^3^, TR = 7 ms, TE = 3 ms, flip angle = 8°, SENSE factor = 2.2, and total scan duration = 5 min 37.9 s. Images were processed in a blinded, automated fashion using FreeSurfer (https://surfer.nmr.mgh.harvard.edu) and FSL (https://web.mit.edu/fsl_v5.0.10/fsl/doc/wiki/FSL.html). Brain tissue volume (i.e., BrainSegNotVent) excluding ventricles, CSF, choroid plexus, and total intracranial volume were computed with FreeSurfer's *recon‐all* pipeline, which included FLAIR images to enhance gray‐white boundary contrast [22]. FLAIR images were collected with the following parameters: FOV = 240 × 240 mm^2^, 48 axial slices, acquired and reconstructed in‐plane resolution = 1 × 1 mm^2^, slice thickness = 3 mm, TR = 11 000 ms, TE = 125 ms, inversion time = 2800 ms, and total scan duration = 6 min 36 s. Total intracranial volume (the total intracranial space) was estimated using FreeSurfer (https://surfer.nmr.mgh.harvard.edu/fswiki/eTIV) based on the T1‐weighted 3D MPRAGE scan acquired on each subject. Intracranial CSF volume was estimated using *FMRIB's Automated Segmentation Tool* in FSL. Brain and CSF volumes were normalized to intracranial volume to correct for individual head size differences. Total cerebral arterial flow rate was normalized to brain mass using a conversion factor of 1.06 g/mL [23].

### 2.6 Physical Activity, VO_2_max, and Left Ventricular Morphometry

Weekly physical activity was quantified using a modified Godin Leisure‐Time Exercise Questionnaire [24] and expressed in metabolic equivalents. VO_2_max was measured in athletes via treadmill testing (ORK‐7000; Ohtaki Root Kogyo, Iwate, Japan) using a breath‐by‐breath analyzer (AE‐310S, Minato Medical Science, Kobe, Japan). VO_2_max was confirmed by meeting at least two of the following criteria: (1) respiratory quotient ≥ 1.1, (2) HR ≥ 90% of age‐predicted maximum, and (3) Borg scale ≥ 19 [25].

Left ventricular mass and end‐diastolic volume (LVEDV) at rest were assessed in a subset of participants (*n* = 16) using short‐axis CINE MRI with ECG gating and breath‐holding, and analyzed via the modified Simpson's method (QMass v8.1, Medis Medical Imaging Systems, Netherlands). The imaging was performed with retrospective ECG gating and end‐expiratory breath holding with the following parameters: field of view = 340 × 340 mm, slice thickness = 8 mm, number of slices = 15 with no gap, acquired in‐plane resolution = 2.97 × 1.48 mm^2^ (reconstructed voxel size = 1.41 × 1.41 mm^2^), 30 cardiac phases, and flip angle = 50°. LV end‐diastolic volume was measured with a modified Simpson's method (QMass version 8.1, Medis Medical Imaging Systems, the Netherlands). Left ventricular morphometry was normalized to body surface area. The methodological details are provided in our previous studies [9, 10].

### 2.7 Sample Size Estimate

Sample size was estimated using G*Power (v3.1, Heinrich Heine Universität Düsseldorf) for a between‐group comparison of cerebral blood flow waveforms. The sample size for this study was initially estimated based on a previous investigation that reported significantly higher aortic compliance in endurance athletes compared with sedentary individuals (effect size = 1.38; two‐sided *α* = 0.05; power [1—*β*] = 0.95; number of groups = 2) [26]. Although no prior study has comprehensively evaluated cerebral blood and CSF flow dynamics in endurance athletes versus sedentary adults, ultrasonography‐based research has demonstrated that athletes have a higher cerebral arterial flow rate than their sedentary counterparts (effect size = 1.19; two‐sided *α* = 0.05; power [1—*β*] = 0.95; number of groups = 2) [27]. Based on these two studies [26, 27], a sample size of 15 participants per group was expected to provide 95% statistical power to detect significant group differences in cerebral arterial flow measures between the athlete and sedentary groups in the present study.

### 2.8 Statistical Analysis

Data normality was assessed using the Shapiro–Wilk test, along with visual inspections of histograms and Q‐Q plots. Depending on the results of normality checks, group comparisons between endurance athletes and sedentary individuals were conducted using either independent *t*‐tests or Mann–Whitney *U* tests. The linear mixed model (LMM) analysis examined the main and interaction effects of group and cardiac phase on total cerebral arterial flow measures and aortic CSA‐TI. Additionally, LMM assessed the effects of group and ROI (i.e., different vessels) on cerebral arterial and venous flow measures. When a significant interaction was found, post hoc pairwise comparisons were conducted using the Bonferroni correction. Pearson's correlation and mediation analyses were performed to explore associations among HR, SV, cerebral blood and CSF flow volumes, aortic CSA‐TI, and brain volumetric measures across all participants. Mediation analysis employed bootstrapping with 5000 samples to calculate bias‐corrected 95% confidence intervals (CI) using the PROCESS macro for SPSS. An indirect effect was considered statistically significant if the 95% CI did not include zero. Statistical significance was set a priori at *p* < 0.05. All results are reported as means ± standard deviations (SD) or 95% CI, as appropriate. All statistical analyses were conducted using SPSS version 25 (IBM Corporation, Armonk, NY, 2011).

## 3 Results

Table 1 shows basic characteristics of athletes and sedentary controls. Both groups were similar in age, height, and BMI, although athletes had significantly lower body mass. As expected, athletes reported markedly higher physical activity, and their VO_2_max exceeded the 90th percentile of the general population for age and sex [28].

**TABLE 1:** Characteristics of the endurance athlete and sedentary groups.

	Athlete (*n* = 15)	Sedentary (*n* = 19)	*p*
	Mean ± SD	Mean ± SD	
Age (years)	20 ± 1	21 ± 2	0.157
Height (cm)	171 ± 6	173 ± 5	0.465
Body mass (kg)	58 ± 6	66 ± 13	**0.016**
Body mass index (kg/m^2^)	20 ± 1	22 ± 4	0.137
Weekly METs (3.5 mL/kg/min)	108 ± 22	14 ± 12	**< 0.001**
VO_2_max (mL/kg/min)	69.5 ± 3.1	—	—
Heart rate (bpm)	53 ± 6	67 ± 12	**< 0.001**
Systolic blood pressure (mmHg)	109 ± 11	116 ± 11	0.071
Diastolic blood pressure (mmHg)	65 ± 9	72 ± 7	**0.014**
Cardiac MRI Measures			
Stroke volume (mL)	97 ± 18	82 ± 11	**0.007**
Stroke volume index (mL/m^2^)	58 ± 9	46 ± 6	**< 0.001**
Cardiac output (L/min)	4.63 ± 0.64	4.70 ± 0.62	0.750
Cardiac index (L/min/m^2^)	2.77 ± 0.35	2.65 ± 0.40	0.391
Left ventricular mass (g)^a^	157 ± 21	124 ± 14	**0.002**
Left ventricular mass index (g/m^2^)^a^	96 ± 16	69 ± 5	**0.009**
LVEDV (mL)^a^	159 ± 22	124 ± 11	**0.001**
LVEDV index (mL/m^2^)^a^	97 ± 12	70 ± 7	**< 0.001**
Brain MRI measures			
Intracranial volume (mL)^b^	1482 ± 199	1523 ± 153	0.512
Total brain volume (mL)^b^	1169 ± 87	1149 ± 68	0.320
Normalized total brain volume (%ICV)^b^	79.7 ± 8.3	76.0 ± 6.9	0.360
CSF volume (mL)^b^	152 ± 19	146 ± 25	0.438
Normalized CSF volume (%ICV)^b^	10.5 ± 2.1	9.7 ± 2.1	0.303

*Note:* Data are shown as mean ± standard deviation (SD). *p* values < 0.05 are bold. VO_2_max was measured only in athletes. Some of the data in this table have been reported in our previous studies [9, 10, 17].

Abbreviations: CSF, cerebrospinal fluid flow; ICV, intracranial volume; LVEDV, left ventricular end‐diastolic volume; METs, metabolic equivalent; SD, standard deviation; VO_2_max, maximal oxygen uptake.

^a^ *n* = 16 (6 athlete, 10 sedentary).

^b^ *n* = 32 (15 athlete, 17 sedentary).

### 3.1 Group Analyses

Compared with sedentary controls, athletes exhibited significantly lower resting HR and greater SV, left ventricular mass, and LVEDV. Athletes also demonstrated greater aortic recoil (5.7 cm^2^·s [4.1–7.3] vs. 4.1 cm^2^·s [2.6–5.6], *p* < 0.05), whereas aortic expansion did not differ significantly between groups (1.8 cm^2^·s [0.1–3.5] vs. 1.6 cm^2^·s [0.4–2.8], n.s.; Figure 2B).

**FIGURE 3:**
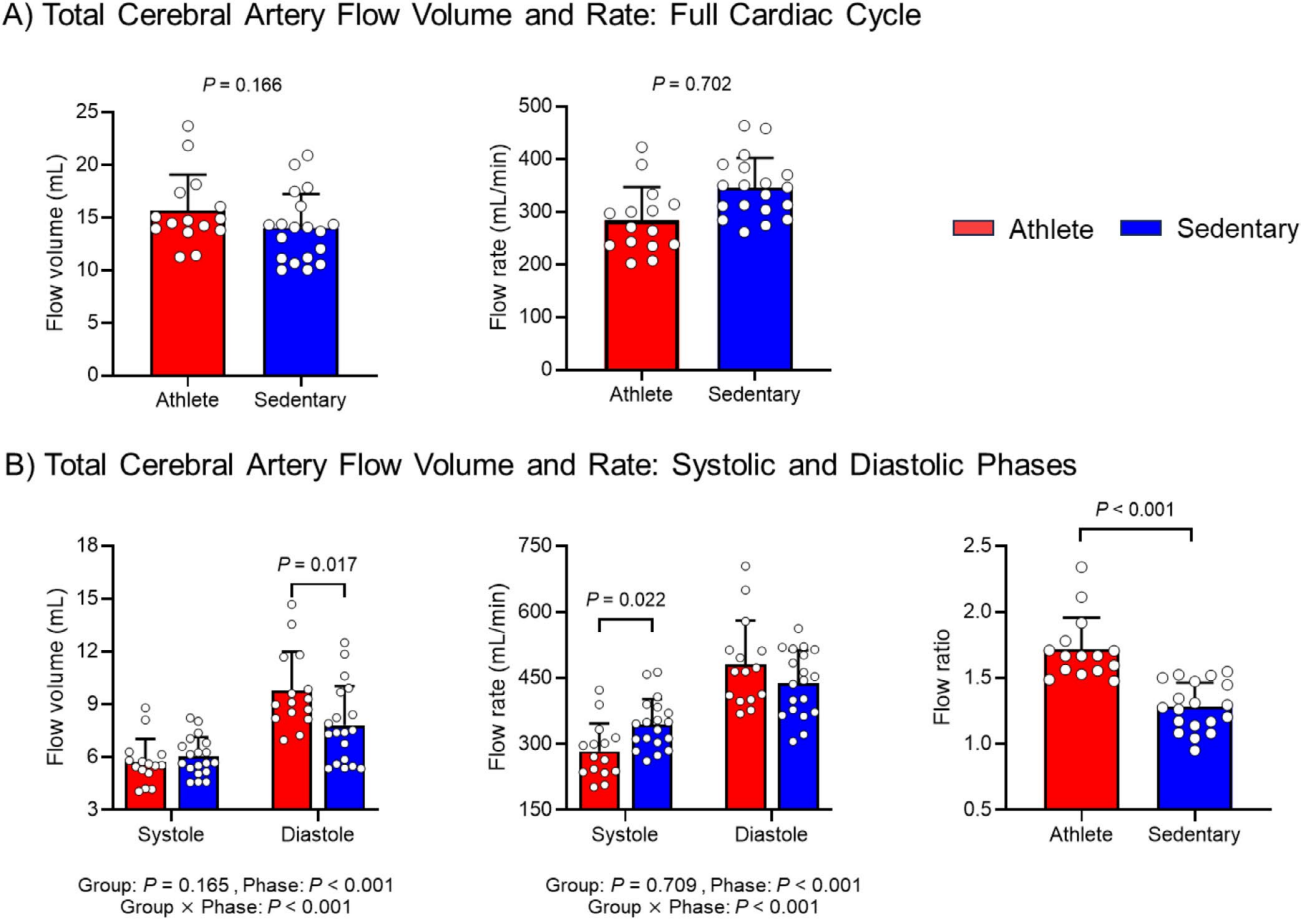
(A) Group comparisons of flow volume (left) and rate (right) in the total cerebral artery (i.e., the sum of internal carotid and vertebral arterial flow) across the full cardiac cycle. (B) Flow volume (left) and rate (middle) in the systolic and diastolic phases, and flow ratio (right) compared between athlete and sedentary groups. Flow ratio was calculated as diastolic flow divided by systolic flow. The use of flow volume or rate yields the same ratio value. *p*‐values were derived from independent *t*‐test and linear mixed model analysis.

Brain tissue, CSF, and intracranial volumes were comparable between groups. Over the full cardiac cycle, total cerebral arterial flow volume and rate were not significantly different between athletes and sedentary controls (volume: 15.6 ± 3.4 vs. 14.0 ± 3.3 mL, *p* = 0.166; rate: 772 ± 158 vs. 791 ± 119 mL/min, *p* = 0.702; Figure 3A). After normalization to brain mass, flow rates remained similar between groups (49.2 ± 7.3 vs. 48.9 ± 8.7 mL/100 g/min, *p* = 0.906).

A significant interaction between group and cardiac phase (systole vs. diastole) was found for both flow volume and rate (Figure 3B). Post hoc test revealed that athletes had greater diastolic flow volume (9.8 mL [3.9–15.7] vs. 7.8 mL [3.0–12.6], *p* = 0.017) and lower systolic flow rate (284 mL/min [255–312] vs. 345 mL/min [323–367], *p* = 0.022). Consequently, the diastolic‐to‐systolic arterial flow ratio was significantly higher in athletes than in the sedentary group (1.72 ± 0.24 vs. 1.28 ± 0.18; *p* < 0.001).

Table 2 summarizes regional arterial and venous flow analyses. Significant main effects of ROI were found for all measurements, indicating regional heterogeneity of blood flow. Over the full cardiac cycle, no significant group effects were found for arterial and venous flow measures. However, phase‐specific analyses revealed a significant group effect for diastolic arterial flow volume, indicating greater diastolic flow volume in athletes than in sedentary individuals. For systolic arterial flow rate, both a significant group effect and group × ROI interaction were found. Post hoc tests revealed that the athlete group had a lower systolic flow rate in the ICA than the sedentary group. Consistent with these results, arterial flow ratios were significantly higher in athletes across all ROIs, indicating a relative volumetric shift toward diastolic flow.

**TABLE 2:** Regional analysis of the arterial and venous flow volume, rate, and ratio compared between the endurance athlete and sedentary groups.

		Athlete (*n* = 15)	Sedentary (*n* = 19)	*p*
		Mean (95% CI)	Mean (95% CI)	
Arterial flow volume (mL)				
Full cardiac cycle	ICA	11.4 (10.4, 12.3)	10.3 (9.4, 11.1)	Group: 0.084
	VA	4.3 (3.6, 4.9)	3.7 (3.1, 4.3)	ROI: **< 0.001**
	BA	3.6 (3.0, 4.1)	3.3 (2.8, 3.8)	Interaction: 0.542
	rMCA	4.1 (3.5, 4.6)	3.4 (2.9, 3.9)	
Systolic phase	ICA	4.2 (3.8, 4.5)	4.4 (4.1, 4.7)	Group: 0.569
	VA	1.6 (1.4, 1.9)	1.6 (1.4, 1.8)	ROI: **< 0.001**
	BA	1.3 (1.0, 1.5)	1.3 (1.1, 1.5)	Interaction: 0.780
	rMCA	1.4 (1.2, 1.6)	1.4 (1.2, 1.6)	
Diastolic phase	ICA	7.1 (6.4, 7.8)	5.8 (5.2, 6.4)	Group: **0.007**
	VA	2.6 (2.2, 3.1)	2.1 (1.7, 2.4)	ROI: **< 0.001**
	BA	2.3 (1.9, 2.7)	1.9 (1.6, 2.3)	Interaction: 0.144
	rMCA	2.6 (2.2, 3.1)	2.0 (1.6, 2.4)	
Arterial flow rate (mL/min)				
Full cardiac cycle	ICA	563 (525, 602)	580 (545, 614)	Group: 0.821
	VA	211 (180, 241)	211 (184, 238)	ROI: **< 0.001**
	BA	174 (148, 200)	181 (157, 204)	Interaction: 0.767
	rMCA	203 (177, 228)	194 (172, 217)	
Systolic phase	ICA	207 (190, 225)	251 (236, 266)*	Group: **0.006**
	VA	80 (67, 92)	94 (82, 105)	ROI: **< 0.001**
	BA	61 (50, 72)	74 (64, 84)	Interaction: **0.034**
	rMCA	70 (57, 82)	80 (69, 91)	
Diastolic phase	ICA	351 (324, 378)	323 (299, 347)	Group: 0.107
	VA	129 (110, 148)	115 (98, 132)	ROI: **< 0.001**
	BA	111 (94, 128)	105 (90, 120)	Interaction: 0.544
	rMCA	131 (112, 150)	113 (96, 130)	
Arterial flow ratio				
Diastolic/systolic	ICA	1.7 (1.6, 1.8)	1.3 (1.2, 1.4)*	Group: **< 0.001**
	VA	1.6 (1.5, 1.7)	1.2 (1.2, 1.3)*	ROI: **< 0.001**
	BA	1.9 (1.7, 2.1)	1.4 (1.3, 1.6)*	Interaction: **< 0.001**
	rMCA	2.0 (1.7, 2.2)	1.4 (1.2, 1.6)*	
Venous flow volume (mL)				
Full cardiac cycle	IJV	−10.5 (−12.7, −8.2)	−8.9 (−11.0, −6.8)	Group: 0.099
	SSS	−7.6 (−8.6, −6.7)	−6.5 (−7.4, −5.7)	ROI: **< 0.001**
	SRS	−2.3 (−2.6, −2.0)	−2.1 (−2.4, −1.8)	Interaction: 0.264
Venous flow rate (mL/min)				
Full cardiac cycle	IJV	−517 (−624, −410)	−489 (−590, −389)	Group: 0.709
	SSS	−373 (−415, −332)	−366 (−402, −329)	ROI: **< 0.001**
	SRS	−113 (−128, −97)	−118 (−132, −104)	Interaction: 0.816

*Note:* Data are shown as mean and 95% confidence interval (95% CI). *p* values < 0.05 are bold. Linear mixed model analyzed the main and interaction effects of group and region‐of‐interest (ROI) on outcomes. Post hoc comparisons were corrected by the Bonferroni method.

Abbreviations: BA, basilar artery; CSF, cerebrospinal fluid; ICA, internal carotid artery; IJV, internal jugular vein; rMCA, right middle cerebral artery; SRS, straight sinus; SSS, superior sagittal sinus; VA, vertebral artery.

* *p* values < 0.05 for post hoc test.

Table 3 presents CSF flow results. All CSF flow parameters were similar between groups.

**TABLE 3:** Cerebrospinal fluid (CSF) flow volume and rate compared between the endurance athlete and sedentary groups.

		Athlete (*n* = 15)	Sedentary (*n* = 19)	*p*
		Mean ± SD	Mean ± SD	
Flow volume (mL)	Cranial	0.09 ± 0.05	0.08 ± 0.04	0.607
	Caudal	−0.10 ± 0.06	−0.09 ± 0.03	0.891
	Net	−0.008 ± 0.015	−0.007 ± 0.014	0.821
	Absolute	0.20 ± 0.11	0.17 ± 0.07	0.607
Flow rate (mL/min)	Cranial	4.58 ± 2.42	4.62 ± 2.14	0.732
	Caudal	−4.95 ± 2.89	−4.97 ± 1.77	0.451
	Net	−0.38 ± 0.72	−0.22 ± 0.81	0.811
	Absolute	9.53 ± 5.29	9.59 ± 3.86	0.560
Peak flow difference (mL/s)		0.51 ± 0.26	0.54 ± 0.21	0.451

*Note:* Data are shown as mean ± standard deviation (SD).

Linear slope analysis showed that the diastolic decay (slope 4) of total cerebral arterial, ICA, and VA waveforms was less steep in athletes compared with sedentary controls (Figure S1 and Table S1). Similarly, the downstroke slope (slope 1) of the SSS waveform was less steep in athletes.

PI showed no significant group effect (*p* = 0.700) or group × ROI interaction (*p* = 0.981), but varied significantly by region (*p* < 0.001, Supplementary Figure 2). Based on the post hoc tests, PI decreased in the following order: VA (1.280 [1.160–1.399]) > ICA (0.998 [0.917–1.079]), rMCA (0.896 [0.818–0.974]), BA (0.884 [0.804–0.964]) > IJV (0.617 [0.505–0.728]) > SSS (0.286 [0.255–0.317]), SRS (0.278 [0.245–0.311]).

### 3.2 Correlation and Mediation Analyses

**FIGURE 4:**
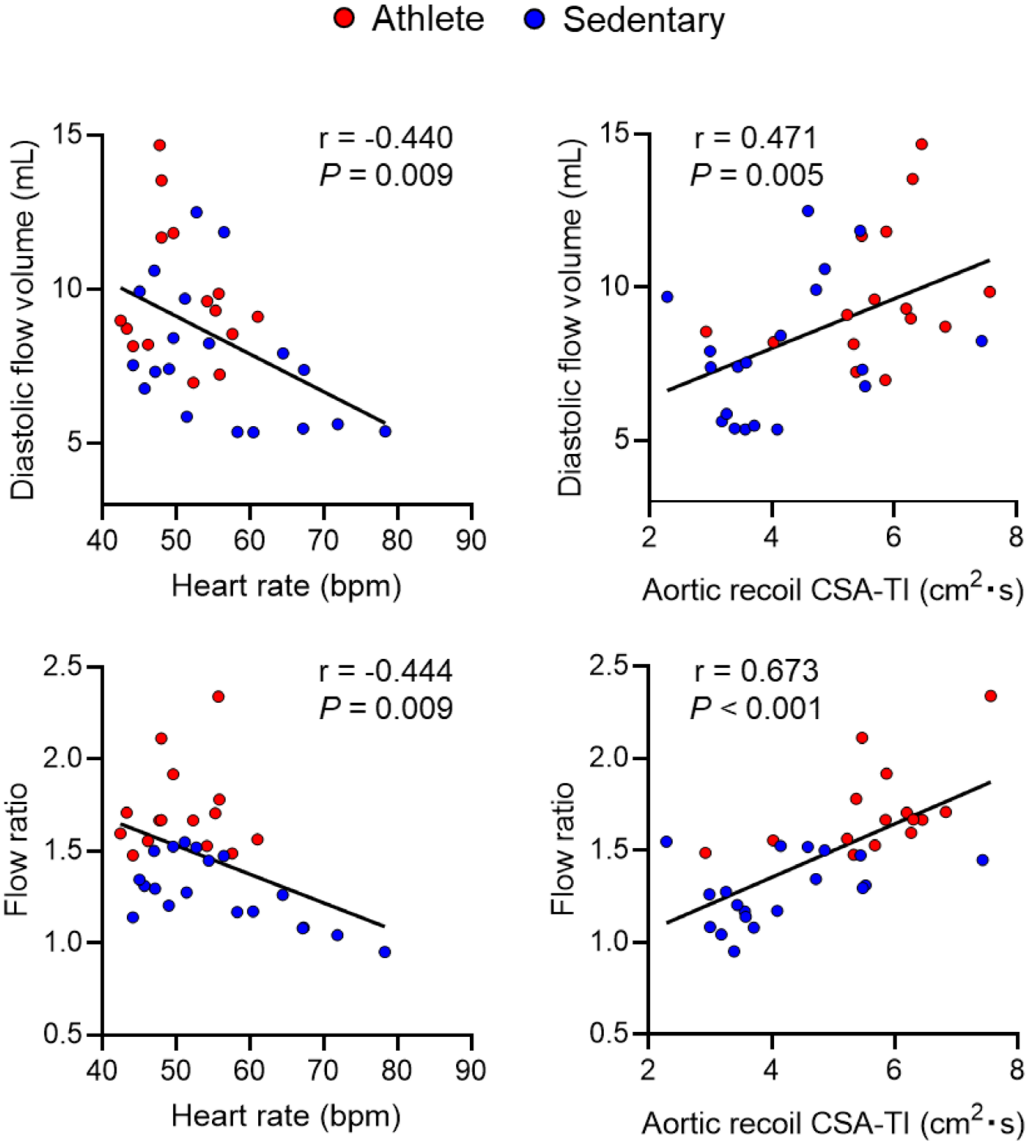
Pearson correlations of heart rate (left) and aortic recoil cross‐sectional area‐time integral (CSA‐TI; right) with diastolic flow volume (top) and flow ratio (bottom) measured from the total cerebral artery (i.e., the sum of internal carotid and vertebral arterial flow) in all participants. Red and blue circles indicate athlete and sedentary participants, respectively. Flow ratio was calculated as diastolic flow divided by systolic flow. The use of flow volume or rate yields the same ratio value.

Across all participants, HR was inversely correlated with greater aortic recoil, diastolic flow volume, and flow ratio of the total cerebral artery (Figure 2C and Figure 4). Aortic recoil correlated positively with diastolic flow volume and flow ratio (Figure 4) and negatively with systolic flow rate of the total cerebral artery (*r* = −0.418, *p* = 0.014). SV was positively correlated with aortic recoil (*r* = 0.562, *p* < 0.001) and expansion (*r* = 0.410, *p* = 0.016), and diastolic flow volume of the total cerebral artery (*r* = 0.442, *p* = 0.009). Intracranial volume was correlated positively with total arterial (*r* = 0.366, *p* = 0.036) and CSF absolute (*r* = 0.395, *p* = 0.025) flow volumes and negatively with IJV flow volume (*r* = −0.400, *p* = 0.028; Table S2).

**FIGURE 5:**
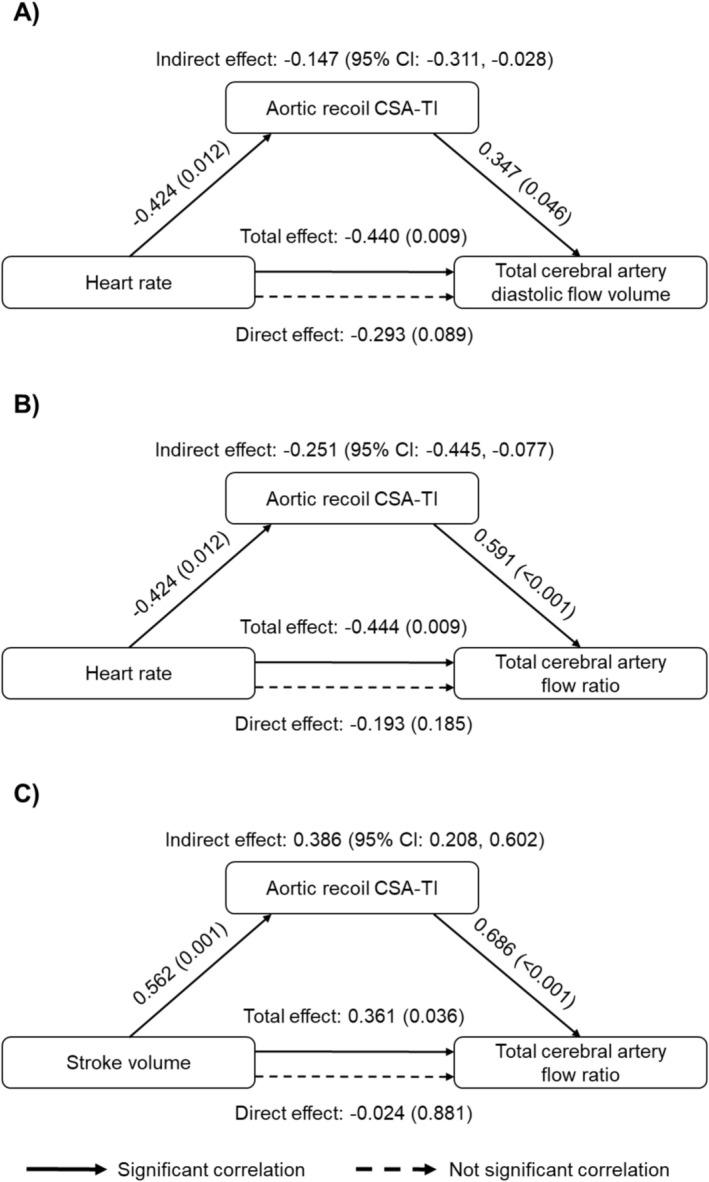
Results of mediation analysis showing that aortic recoil cross‐sectional area‐time integral (CSA‐TI) mediates: (A) the association between heart rate and diastolic flow volume in the total cerebral artery (i.e., the sum of internal carotid and vertebral arterial flow); (B) the association between heart rate and total cerebral artery flow ratio; and (C) the association between stroke volume and total cerebral artery flow ratio in all participants. Standardized coefficients and (*p*‐values) are reported for total and direct effects; standardized coefficients and 95% confidence intervals (CIs) are shown for the indirect effect. Flow ratio was calculated as diastolic flow divided by systolic flow. The use of flow volume or rate yields the same ratio value.

Mediation analysis revealed that the association between slower HR and higher diastolic flow volume of the total cerebral artery was mediated by increased aortic recoil (Figure 5A). Moreover, aortic recoil significantly mediated the associations of HR (Figure 5B) and SV (Figure 5C) with flow ratio of the total cerebral artery.

## 4 Discussion

This study investigated how endurance training‐induced cardiovascular adaptations relate to cerebral blood and CSF flow profiles in young male endurance athletes using CINE PC‐MRI. The main findings are as follows. First, compared with sedentary controls, endurance athletes showed enhanced aortic recoil along with lower resting HR and higher SV. Second, athletes exhibited greater diastolic cerebral arterial flow volume and lower systolic flow rate, despite no group differences in total flow volume or rate across the full cardiac cycle. Third, aortic recoil mediated the relationship between lower HR and increased diastolic cerebral arterial flow volume, an effect also supported by the elevated diastolic‐to‐systolic flow ratio. Finally, total cerebral arterial flow volume was associated with both venous and CSF flow volumes across the full cardiac cycle, as well as with intracranial volume. Collectively, these findings suggest that enhanced aortic recoil in endurance athletes increases the diastolic cerebral arterial flow volume without increasing the systolic or total flow rate, which may improve the efficiency of brain perfusion with fewer cardiac cycles.

Our athlete group exhibited hallmark features of the “athlete's heart,” including lower resting HR and higher SV, LVEDV, and left ventricular mass, consistent with previous studies [8]. Although cerebral blood and CSF flow volumes across the full cardiac cycle were similar between groups, waveform analysis revealed a higher diastolic‐to‐systolic arterial flow ratio in endurance athletes than in sedentary controls. Notably, ascending aortic recoil emerged as a key physiological mechanism, mediating the association between reduced HR and greater diastolic cerebral arterial flow volume in all participants. These results support the concept that cardiovascular adaptations from endurance training interact with cerebrovascular hemodynamics while preserving total arterial inflow magnitude. A prior study demonstrated a strong correlation between lower HR and longer diastolic duration in healthy young and older adults [29], while age‐related aortic stiffening was associated with reductions in cerebral diastolic perfusion [30]. In addition, a recent modeling study of cerebral arteriovenous pressure transmission suggests that a greater Windkessel time constant may buffer pulsatile energy and sustain diastolic flow in individuals with bradycardia [31]. Our findings extend this literature by showing that enhanced aortic recoil is accompanied by elevated diastolic cerebral arterial volume and reduced systolic flow rate, a temporal redistribution that may support steady cerebral perfusion dynamics during prolonged cardiac cycles in endurance athletes.

Despite the markedly higher SV observed in the athlete group, the systolic cerebral blood flow rate in the ICA (the main conduit of cerebral perfusion) was lower compared with sedentary controls. This apparent paradox likely results from the interplay between slower HR and similar systolic flow volume between groups. Notably, SV was not associated with systolic flow volume but correlated positively with diastolic flow volume, aortic recoil, and aortic expansion. These associations suggest that greater SV in athletes, combined with enhanced aortic Windkessel function, shifts flow volume toward the diastolic phase. These aortic Windkessel effects, which attenuate systolic flow rate and augment diastolic flow volume [11], may thereby mitigate excessive cerebral pulsatility while maintaining steady perfusion, offering protection for delicate neural tissues in the brain.

According to the Monro‐Kellie doctrine, the total intracranial volume comprising brain tissue, blood, and CSF remains constant, thereby stabilizing intracranial pressure [12]. In the current study, we found no group differences in total brain, CSF, or blood flow measures across the full cardiac cycle, despite clear phase‐specific shifts in the arterial flow volume and rate. Furthermore, total cerebral arterial flow volume was positively correlated with intracranial volume and absolute CSF flow volume, and negatively with venous flow volume, suggesting an interaction between arterial inflow and compensatory venous and CSF outflow volumes during the cardiac cycle. These findings are supported by a previous study in healthy young adults showing that changes in intracranial arterial volume elicit an inverse response in venous and CSF volumes [32]. Our findings provide additional insight beyond a prior study, indicating that aerobic training does not alter the overall interaction of intracranial fluids, while its temporal characteristics may be modulated through cardiovascular adaptations such as slower HR, increased SV, and enhanced aortic recoil. Therefore, endurance training appears to modify the timing and distribution of intracranial fluid flow measures rather than its overall magnitude.

Previous studies using transcranial Doppler (TCD) ultrasound have reported higher mean cerebral blood velocity [33] and PI [34] in endurance‐trained adults compared with sedentary controls. However, we found no group differences in cerebral arterial flow volume and rate or PI when measured across the full cardiac cycle using CINE PC‐MRI. These discrepancies likely stem from methodological differences: TCD offers high temporal resolution but measures velocity in major cerebral vessels, while CINE PC‐MRI quantifies volumetric flow measures simultaneously across multiple vessels (e.g., ICA, VA, IJV) with lower temporal resolution. Irrespective of groups, our results demonstrated a gradual reduction in PI from extracranial arteries (VA, ICA) to intracranial arteries (rMCA, BA), extracranial (IJV) and intracranial veins (SSS, SRS). This arterial‐to‐venous PI gradient likely reflects the damping effects of vessel wall properties, brain tissue compliance, and CSF flow resistance. Specifically, pressure and flow pulsatility generated by left ventricular ejection is transmitted to low‐resistance cerebral circulation, elevating arterial PI, whereas downstream arteriolar and microvascular resistance reduces pulsatile amplitude within the venous circulation. The markedly low PI in SSS and SRS may reflect their semirigid dural boundaries, which limit sinus wall expansion, while upstream arterial elasticity and CSF/brain compliance dissipate most cardiac‐ and ICP‐derived pulsatile energy before the residual pulsatility is expressed as oscillations in the sinus flow waveform. This gradient diminishes with aging and may converge between the arterial and venous PIs in older adults [19]. Because cerebral pulsatility is integral to both nutrient delivery and waste clearance via blood and CSF flow, understanding how pulsatility is altered by endurance training may yield insights into protective mechanisms against cerebrovascular pathology.

The cerebral blood and CSF flow values obtained in the present study were within the physiological ranges reported for healthy young adults [18, 35, 36]. Arterial (ICA: 273–299 mL/min, VA: 95–116 mL/min, BA: 159–195 mL/min, rMCA: 179–218 mL/min) and venous flow rates (IJV: 430–577 mL/min, SSS: 342–397 mL/min, SRS: 105–126 mL/min) were comparable to those reported in prior studies evaluating cerebral blood flow in healthy young adults (ICA: 276 ± 47 mL/min, VA: 109 ± 23 mL/min, BA: 162 ± 44 mL/min, rMCA: 161 ± 31 mL/min, IJV: 449 ± 173 mL/min, SSS + SRS: 478 ± 94 mL/min) [35, 36]. Our aqueductal CSF flow volumes (cranial: 0.08–0.09 mL/beat; caudal: 0.09–0.10 mL/beat) were slightly higher than prior reports but nevertheless remained within the ranges observed in healthy young adults scanned on a 3 T Philips MRI system in the supine position (0.01–0.12 mL/beat) [18]. Differences across studies may stem from variations in imaging sequences, MRI system (e.g., hardware, field strength), and biological and physiological factors such as age, sex, heart rate, and respiration, which are known to modulate cerebral blood and CSF flow characteristics.

### 4.1 Methodological Considerations

This study has several limitations. First, a cross‐sectional design cannot draw causal inference, although studying endurance athletes offers a valuable model for examining the chronic effects of sustained aerobic training on cerebral blood and CSF flow measures in relation to cardiovascular adaptations. Second, only male athletes were recruited during the experimental period, although we also attempted to recruit female athletes. Future research should investigate potential sex‐specific differences in cerebrovascular responses to endurance training because females are shown to have a higher cerebral arterial flow rate than males [30], which may alter the venous and CSF flow profile. Third, VO_2_max was assessed only in the athlete group, limiting the ability to assess associations between aerobic fitness and cerebral blood flow parameters across the entire sample. Fourth, end‐tidal carbon dioxide was not measured in the current study, which may have affected the resting cerebral blood and CSF flow measures. Fourth, all measurements were obtained under resting supine conditions; subsequent studies should explore how cerebral blood and CSF flow measures vary with changes in posture or during dynamic exercise. Finally, stationary‐tissue ROIs were not used for phase‐offset correction, which may leave modest residual background phase offsets particularly for CSF flow with slow velocities, but this is not likely to alter cardiac phase timing or between‐group phase‐specific comparisons of arterial flow measurements.

Despite these limitations, this study has several strengths. To our knowledge, this is the first study investigating the associations of cardiovascular adaptations from endurance training with cerebral blood and CSF flow characteristics using the aortic and cerebral CINE PC‐MRI. This comprehensive, multimodal approach can deepen our understanding of the heart‐brain interactions in the context of endurance training. Our findings also offer a physiological reference for future studies investigating how aerobic exercise training may help preserve brain function and prevent age‐related disorders such as Alzheimer's disease and related dementias [37]. Furthermore, our data provide empirical support for the Monro‐Kellie hypothesis, demonstrating that the dynamic interactions among brain tissue, blood, and CSF are maintained across the cardiac cycle, even with significant cardiovascular adaptations.

## 5 Perspective

Endurance athletes with slower HR and higher SV and aortic recoil exhibited similar cerebral blood and CSF flow volumes and rates across the full cardiac cycle as sedentary controls, but demonstrated a distinct redistribution of cerebral arterial flow volume toward the diastolic phase. Furthermore, enhanced aortic recoil mediated the association between slower HR and increased diastolic cerebral flow volume, consistent with the elevated diastolic‐to‐systolic flow ratio. Collectively, these findings suggest that cardiovascular adaptations to endurance training facilitate more efficient cerebral perfusion with fewer cardiac cycles by augmenting diastolic flow volume and attenuating systolic flow rate. These results provide a physiological foundation for understanding how aerobic training promotes efficient heart‐brain coupling and may help protect against age‐related cerebrovascular decline. Beyond the role of aortic recoil in cerebral hemodynamics, prior studies have shown that aerobic training improves respiratory muscle function and modifies chemoreflex sensitivity. These adaptations have also been proposed as potential modulators of cerebral blood and CSF flow dynamics [38, 39] and may contribute to efficient energy supply and waste clearance [40]. Future longitudinal and interventional studies are warranted to determine how exercise‐induced improvements in aortic, respiratory, and autonomic functions translate to cerebral hemodynamic regulation and brain health across the lifespan.

## Funding

This study was supported by the Japan Society for the Promotion of Science (24KJ2216, DH; 23K24748, 25K21803, TT). The ARIHHP Cooperative Grant (University of Tsukuba).

## Conflicts of Interest

The authors declare no conflicts of interest.

## Supporting information


**Data S1:** sms70261‐sup‐0001‐Supinfo.docx.

## Data Availability

The data that support the findings of this study are available on request from the corresponding author. The data are not publicly available due to privacy or ethical restrictions.
