# Supplementary material for: Cerebral Blood and Cerebrospinal Fluid Flow Dynamics in Endurance Athletes: Associations With Aortic Recoil and Heart Rate
Source: Scand J Med Sci Sports. 2026 Mar 12;36(3):e70261. doi: 10.1111/sms.70261 (PMC12980292; doi:10.1111/sms.70261)
Supplement: Supplementary file 1 — Data S1: sms70261‐sup‐0001‐Supinfo.docx. [file SMS-36-e70261-s001.docx]

**Supplementary Methods**

*Rationale for region of interest (ROI) selections*

Extracranial arterial inflow was measured bilaterally from the internal carotid (ICA) and vertebral (VA) arteries, which perfuse the entire brain through the anterior and posterior circulations, respectively. These two arteries are estimated to contribute approximately 75% and 25% of the total cerebral blood flow (1). The primary extracranial venous outflow was measured from the bilateral internal jugular veins (IJV). *Intracranial* arterial flow was measured from the right middle cerebral artery (rMCA) and basilar (BA) arteries. The MCA, one of the terminal branches of the ICA, primarily supplies the anterior circulation and also extensive areas of the cerebrum. Compared to the anterior and posterior cerebral arteries, the MCA delivers about 2 to 2.5 times the amount of blood flow, reflecting the majority of intracranial hemodynamics (2). The BA is formed by the union of the VAs inside the cranium, supplies the posterior circulation including the occipital lobe, brainstem, and cerebellum, and serves as an important vessel reflecting the posterior cerebral circulation. Intracranial venous flow was measured from the superior sagittal (SSS) and straight (SRS) sinuses in the occipital area. The SSS collects venous blood from the outer and superficial regions of the cerebral hemispheres, while the SRS drains venous blood from the deeper structures of the brain, with both ultimately emptying into the internal jugular vein (IJV) (3). CSF flow was measured from the cerebral aqueduct of Sylvius, the primary route connecting the third and fourth ventricles (4). Its narrow, well-defined structure enables reliable quantification of cardiac-driven, bidirectional CSF pulsations that may reflect intracranial compliance and pressure transmission. Bilateral data were summed to reduce the number of comparisons. This study examined the impact of aerobic exercise-related cardiovascular adaptations on cerebral fluid dynamics in endurance athletes by measuring extra- and intracranial blood and CSF flow in major pathways across the brain.

**Supplementary Results**

| **Supplementary Table 1.** Slope analysis of the brain blood and cerebrospinal fluid (CSF) flow waveforms in the endurance athlete and sedentary groups | | | | | | | | |
| --- | --- | --- | --- | --- | --- | --- | --- | --- |
|  |  | Athlete (N = 15) | | | Sedentary (N = 19) | | | *P* value |
|  |  | mean | ± | SD | mean | ± | SD |  |
| *Arterial Flow* | | | | | | | | |
| Total cerebral artery | Slope 1 | 103 | ± | 35 | 102 | ± | 38 | 0.935 |
| (ICA+VA) | Slope 2 | -32 | ± | 10 | -40 | ± | 14 | 0.053 |
|  | Slope 3 | 23 | ± | 14 | 28 | ± | 10 | 0.291 |
|  | Slope 4 | -9.2 | ± | 2.2 | -11.1 | ± | 2.7 | **0.025** |
| ICA | Slope 1 | 71 | ± | 23 | 69 | ± | 22 | 0.765 |
|  | Slope 2 | -24 | ± | 8 | -28 | ± | 9 | 0.207 |
|  | Slope 3 | 21 | ± | 9 | 19 | ± | 11 | 0.837 |
|  | Slope 4 | -6.5 | ± | 2.0 | -8.2 | ± | 2.0 | **0.012** |
| VA | Slope 1 | 35 | ± | 16 | 36 | ± | 19 | 0.855 |
|  | Slope 2 | -12 | ± | 5 | -15 | ± | 6 | 0.077 |
|  | Slope 3 | 11 | ± | 7 | 12 | ± | 7 | 0.837 |
|  | Slope 4 | -2.6 | ± | 1.0 | -3.3 | ± | 1.0 | **0.015** |
| BA | Slope 1 | 19 | ± | 8 | 23 | ± | 13 | 0.391 |
|  | Slope 2 | -5.9 | ± | 2.8 | -7.6 | ± | 5.8 | 0.336 |
|  | Slope 3 | 5.6 | ± | 2.7 | 7.0 | ± | 5.2 | 0.891 |
|  | Slope 4 | -2.0 | ± | 0.6 | -2.4 | ± | 1.1 | 0.319 |
| rMCA | Slope 1 | 26 | ± | 12 | 21 | ± | 7 | 0.493 |
|  | Slope 2 | -7.5 | ± | 4.1 | -7.0 | ± | 2.0 | 0.973 |
|  | Slope 3 | 6.7 | ± | 3.2 | 6.1 | ± | 2.5 | 0.528 |
|  | Slope 4 | -2.5 | ± | 0.6 | -2.9 | ± | 0.9 | 0.089 |
| *Venous Flow* | | | | | | | | |
| IJV | Slope 1 | -16 | ± | 13 | -20 | ± | 17 | 0.411 |
|  | Slope 2 | 8.8 | ± | 6.1 | 7.3 | ± | 6.0 | 0.370 |
| SSS | Slope 1 | -3.5 | ± | 1.5 | -7.4 | ± | 5.1 | **0.017** |
|  | Slope 2 | 2.8 | ± | 0.9 | 2.3 | ± | 0.8 | 0.093 |
| SRS | Slope 1 | -1.5 | ± | 1.3 | -2.6 | ± | 2.5 | 0.251 |
|  | Slope 2 | 0.8 | ± | 0.5 | 1.0 | ± | 1.1 | 0.758 |
| *Cerebrospinal Fluid Flow* | | | | | | | | |
| CSF | Slope 1 | -1.5 | ± | 0.8 | -2.0 | ± | 0.9 | 0.066 |
|  | Slope 2 | 0.6 | ± | 0.3 | 0.7 | ± | 0.3 | 0.286 |
| Data are shown by mean and standard deviation (SD). Independent t-test was used to compare between the two groups. *P* values < 0.05 are bolded. BA, basilar artery; CSF, cerebrospinal fluid; ICA, internal carotid artery; IJV, internal jugular vein; rMCA, right middle cerebral artery; SRS, straight sinus; SSS, superior sagittal sinus; VA, vertebral artery | | | | | | | | |

| **Supplementary Table 2.** Simple correlations among brain blood flow, cerebrospinal fluid (CSF) flow, brain structure, and cardiovascular measures in all participants | | | | | | | | |
| --- | --- | --- | --- | --- | --- | --- | --- | --- |
|  | Total cerebral artery flow volume | IJV flow volume | CSF absolute flow volume | Intracranial volume | Total brain volume | CSF volume | Stroke volume | Heart rate |
| Total cerebral artery |  | **-0.702** | **0.355** | **0.481** | **0.366** | -0.143 | **0.399** | **-0.369** |
| flow volume |  | **(< 0.001)** | **(0.039)** | **(0.005)** | **(0.036)** | (0.442) | **(0.020)** | **(0.032)** |
| IJV flow volume |  |  | **-0.386** | **-0.400** | -0.208 | -0.269 | **-0.392** | 0.174 |
|  |  |  | **(0.029)** | **(0.028)** | (0.262) | (0.158) | **(0.027)** | (0.341) |
| CSF absolute flow volume |  |  |  | **0.395** | 0.344 | 0.010 | 0.310 | -0.336 |
|  |  |  |  | **(0.025)** | (0.050) | (0.958) | (0.074) | (0.052) |
| Intracranial volume |  |  |  |  | **0.559** | -0.083 | -0.003 | -0.163 |
|  |  |  |  |  | **(0.001)** | (0.658) | (0.988) | (0.374) |
| Total brain volume |  |  |  |  |  | -0.006 | 0.207 | -0.274 |
|  |  |  |  |  |  | (0.976) | (0.249) | (0.123) |
| CSF volume |  |  |  |  |  |  | 0.222 | 0.209 |
|  |  |  |  |  |  |  | (0.230) | (0.259) |
| Stroke volume |  |  |  |  |  |  |  | -0.284 |
|  |  |  |  |  |  |  |  | (0.103) |
| Heart rate |  |  |  |  |  |  |  |  |
|  |  |  |  |  |  |  |  |  |
| Values are Pearson’s correlation coefficients and p-values in parentheses. *P* < 0.05 are bolded. IJV flow volume was included as it represents the primary venous route in the supine resting condition. CSF, cerebrospinal fluid; IJV, internal jugular vein | | | | | | | | |

**
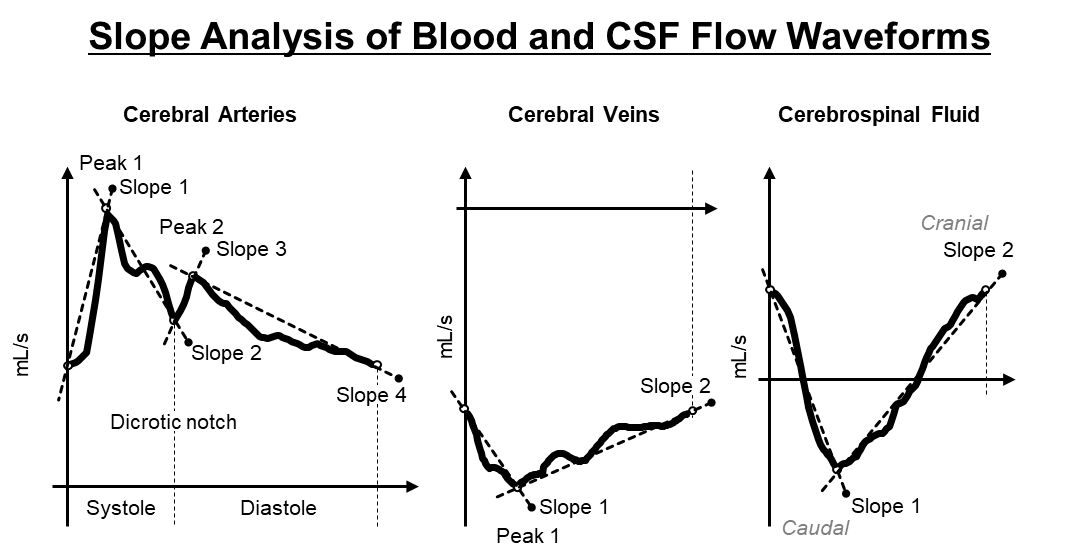
Supplemental Figure 1.** Slope analysis of blood and CSF flow waveforms. Four slopes were calculated from arterial waveforms, and two from venous and CSF waveforms (see text for details). For arterial waveforms, the following slopes were calculated: 1) systolic upstroke: onset to Peak 1, 2) systolic downstroke: Peak 1 to dicrotic notch, 3) diastolic upstroke: dicrotic notch to Peak 2, and 4) diastolic decay: Peak 2 to end of cycle. For the venous and CSF waveforms where dicrotic notch and Peak 2 are absent, the following two slopes were calculated: 1) downstroke: onset to Peak 1 and 2) upstroke: Peak 1 to end of cycle. Peak 1 represents the first maximal flow value after the systolic onset, and Peak 2 (arterial only) was the second maximal flow value after the dicrotic notch. CSF: cerebrospinal fluid


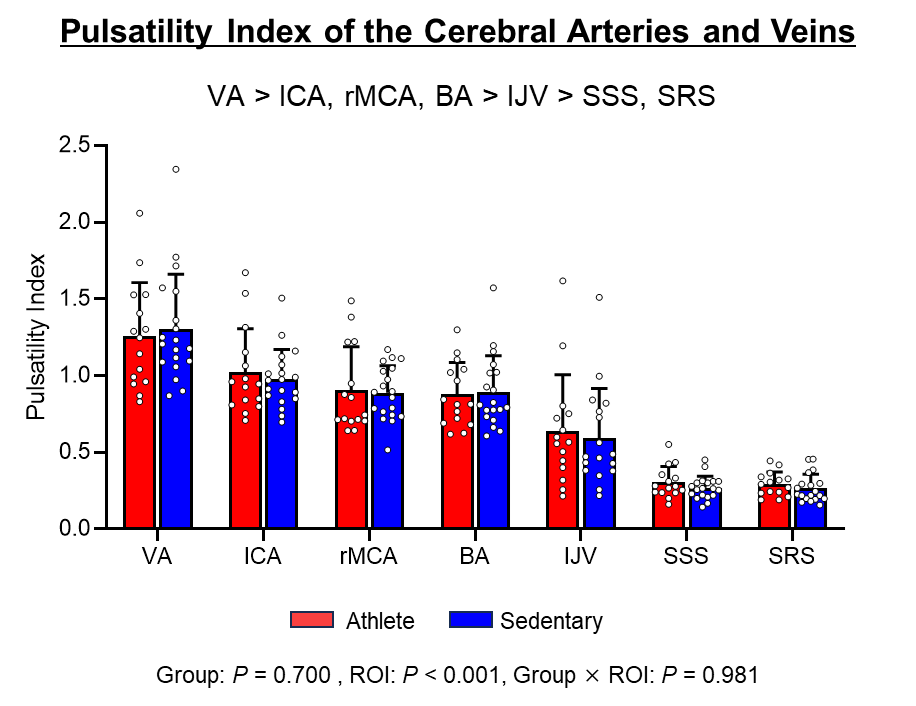
**Supplementary Figure 2.** Pulsatility index of the cerebral arteries and veins compared between the endurance athlete and sedentary groups. Athlete and sedentary groups had similar pulsatility index with regional differences: highest in extracranial arteries (VA, ICA) followed by intracranial arteries (rMCA, BA), extracranial veins (IJV), and intracranial veins (SSS, SRS). BA, basilar artery; ICA, internal carotid artery; IJV, internal jugular vein; rMCA, right middle cerebral artery; ROI: region-of-interest; SRS, straight sinus; SSS, superior sagittal sinus; VA, vertebral artery

**Reference**

1. Schöning M, Walter J, Scheel P. Estimation of cerebral blood flow through color duplex sonography of the carotid and vertebral arteries in healthy adults. Stroke. 1994;25(1):17-22. Epub 1994/01/01. doi: 10.1161/01.str.25.1.17. PubMed PMID: 8266366.

2. Roberts GS, Peret A, Jonaitis EM, Koscik RL, Hoffman CA, Rivera-Rivera LA, et al. Normative Cerebral Hemodynamics in Middle-aged and Older Adults Using 4D Flow MRI: Initial Analysis of Vascular Aging. Radiology. 2023;307(3):e222685. Epub 2023/03/22. doi: 10.1148/radiol.222685. PubMed PMID: 36943077; PubMed Central PMCID: PMCPMC10140641

3. Kiliç T, Akakin A. Anatomy of cerebral veins and sinuses. Front Neurol Neurosci. 2008;23:4-15. Epub 2007/11/16. doi: 10.1159/000111256. PubMed PMID: 18004050.

4. Damkier HH, Brown PD, Praetorius J. Cerebrospinal fluid secretion by the choroid plexus. Physiol Rev. 2013;93(4):1847-92. Epub 2013/10/19. doi: 10.1152/physrev.00004.2013. PubMed PMID: 24137023.
